# Blood Pressure Time in Target Range and its Impact on Clinical Outcomes

**DOI:** 10.1007/s11886-024-02111-9

**Published:** 2024-08-14

**Authors:** Astefanos Al-Dalakta, Chadi Tabaja, Issam Motairek, Abdel Hadi El Hajjar, Neel Agarwal, Julie St. John, Luke J. Laffin

**Affiliations:** 1 Department of Internal Medicine, Cleveland Clinic, 9500 Euclid Avenue, Cleveland, OH 44195 USA; 2 Case Western Reserve University, 10900 Euclid Avenue, Cleveland, OH 44106 USA; 3 Cleveland Clinic, C5Research, 9500 Euclid Avenue, Cleveland, OH 44195 USA; 4 Section of Preventive Cardiology and Rehabilitation, Department of Cardiovascular Medicine, Cleveland Clinic Foundation, 9500 Euclid Avenue, Mail Code JB1, Cleveland, OH 44195 USA

**Keywords:** Hypertension, Time in target range, Time in therapeutic range, Cardiovascular disease, Cardiovascular disease prevention, Cardiovascular outcomes

## Abstract

**Purpose of Review:**

To examine the concept of time in target range for blood pressure (BP) management, exploring its calculation methods, implications for patient outcomes, and potential use in patient care.

**Recent Findings:**

Recent post-hoc analyses of clinical trials and observational studies highlight the importance of BP time in target range in predicting cardiovascular outcomes. Higher time in target range correlates with reduced risks of major adverse cardiovascular events including heart failure, stroke, myocardial infarction and all-cause mortality. Additionally, longer time in target range decreases the risk of incident atrial fibrillation and risk of developing dementia.

**Summary:**

BP time in target range is a novel metric offering valuable insights into BP control and its impact on clinical outcomes. Higher time in target range is consistently associated with better cardiovascular outcomes across various patient populations. However, the clinical application of BP time in target range requires further investigation through prospective clinical trials and real-world studies. Integrating wearable devices for continuous BP monitoring could enhance the practical utility of BP time in target range in hypertension management.

## Introduction

Hypertension is a leading cause of morbidity and mortality worldwide [1]. Despite numerous public awareness initiatives, guideline statements, and easily accessible medications, the rates of blood pressure (BP) control are suboptimal and worsening [2]. Only 54% of adults with hypertension are diagnosed, 42% undergo treatment, and just 21% have their condition under control [3]. Patients with hypertension are at an increased risk of all-cause mortality and increased risk of coronary artery disease, cerebrovascular disease, and heart failure (HF) [4, 5]. Typically, a patient’s BP control is assessed using a BP measurement from the most recent office visit. Although intensive lowering of *office* systolic BP reduces cardiovascular (CV) events and mortality, BP is a continuous variable that fluctuates and can be very different on a day-to-day or month-to-month basis [6]. Gauging longitudinal BP control can be difficult when relying solely on an individual BP measurement, be it in an office or self-monitored setting. Additionally, various studies indicate that coronary artery disease progression, CV mortality, and all-cause mortality are associated with greater visit-to-visit BP variability [7, 8].

Time in target range (TTR) is emerging as a novel metric for BP assessment and offers a unique way to evaluate BP control. TTR refers to the proportion of time during which a patient maintains BP within a predefined range, thereby providing insight into the degree of BP control. The concept of TTR has been used to assess anticoagulation control by monitoring International Normalized Ratio (INR) values in patients treated with warfarin for various clinical indications [9–11]. Higher TTR is associated with reduced risk of thromboembolic events and bleeding complications [10, 11]. The aim of this review article is to examine the concept of TTR for BP management, exploring its calculation methods, implications for patient outcomes, and potential use for patient care.

## How to Assess Time in Target Range

The traditional method for calculating TTR is a straight-forward process. Data is collected, and a target range is established. Each measurement is scrutinized to see if it falls within the established range. The TTR is calculated by summing the durations during which the measurements are within the target range and dividing this sum by the total monitoring period. This gives the percentage of time spent within the target range [12].

Frits R. Rosendaal developed an alternative method, the Rosendaal linear interpolation (RLI) method, which is an established alternative approach for calculating the TTR [13]. It was first used in anticoagulation therapy management and adapted for BP TTR assessment. This technique involves linear interpolation between successive BP measurements to determine the fraction of time spent within the predefined range. Initially, a sequence of BP readings is collected over a specified period for an individual. Following this, consecutive pairs of readings are selected to define intervals that represent fluctuations in BP. Linear interpolation is then applied to calculate the proportion of time each interval falls within the target range. These calculated proportions are aggregated to compute the overall TTR for the assessment period. The RLI method offers a practical and broadly recognized means to evaluate the efficacy of BP management over time [12, 14–16].

## Impact on Cardiovascular Outcomes among Clinical Trial Participants

No large clinical trials pre-specify BP TTR as a subgroup to be analyzed, thus the data associating BP TTR using clinical trial participant data consists of post-hoc analyses. Buckley et al. investigated systolic BP TTR in relation to adverse kidney and CV events using data from the Systolic Blood Pressure Intervention Trial (SPRINT) and the Action to Control Cardiovascular Risk in Diabetes (ACCORD) study. In both studies, participants were assigned to intensive (< 120 mm Hg) or standard (< 140 mm Hg) BP targets. Systolic BP TTR was assessed through RLI using a range of 110–130 mm Hg for the intensive arm and 120–140 mm Hg for the standard arm. Individuals were divided into tertiles of TTR 0%-43%, 43–70% and 70%-100%. The analysis demonstrated a decrease in major adverse cardiovascular events (MACE), stroke, HF hospitalization, CV death as BP time spent in target range increased. In the fully adjusted model, compared to a TTR of 0%, there was 35% reduction of MACE with TTR of 71–100%, additionally there was a linear risk reduction seen with increasing TTR tertiles [17].

Similarly, Fatani et al.'s post hoc analysis of SPRINT demonstrated a significant relationship in individuals with extended periods within the target range. Systolic BP TTR was assessed through RLI using a range of 110–130 mm Hg for the intensive arm and 120–140 mm Hg for the standard arm. Patients were divided into 4 groups of TTR: 0 to < 25%, 25 to < 50%, 50 to < 75%, 75 to 100%. Patients were followed up for a median of 3.3 years. For each standard deviation increase in TTR there was an associated decrease in the risk of a first MACE, with an adjusted hazard ratio (HR) of 0.78 (95% CI: 0.70–0.87). This relationship persisted even after controlling for mean systolic BP and systolic BP variability [16].

Additionally, a secondary analysis of ACCORD examined the effects of TTR on various CV outcomes including MACE, stroke, myocardial infarction (MI), CV death, and heart failure. Participants were followed for an average of 4.9 years and systolic TTR was 110–130 mm Hg. It was observed that TTR of 61–100% compared to 0–22.9% was associated with a reduction of 46% in MACE, 81% in stroke, 53% in HF, 33% in MI, 37% in CV death and 30% in all-cause mortality [18].

## Impact on Outcomes among Observational Study Participants

A recently conducted study evaluated the relationship between long-term BP TTR and CV outcomes in an elderly hypertensive Chinese population. They included 943 patients aged over 75 years, initially recruited in early 2006 and were followed up for a period of 15 years. Participants had at least 3 (and an average of 6) BP measurements to calculate TTR. TTR, defined in this study as systolic BP between 120 and 140 mm Hg, was determined using RLI. The primary endpoint of the investigation was a composite of the initial occurrence of a CV event including MI, stroke, angina, cardiac death, assessed during annual visits. Using adjusted analysis, patients in the highest quartile of TTR demonstrated a 58% lower risk of the composite outcome when compared to the lowest quartile [HR 0.42 (95% CI: 0.29–0.62)]. Furthermore, a 25% reduction in the risk of MACE was observed per standard deviation increase in TTR, with a HR of 0.75 (95% CI: 0.67–0.83) [19].

Doumas et. al evaluated the effect of systolic BP TTR on all-cause mortality among hypertensive patients using data from 689,051 veterans over an average follow-up period of 10 years. TTR was defined as the percentage of systolic BP measurements that fell within 120-140 mmHg. Mortality rates were significantly lower among hypertensive patients who maintained systolic BP within the therapeutic range > 75% of the time, with a mortality rate of 6.5%, compared to 15.6% and 23.5% in those with SBP control between 25–50% and less than 25% of the time, respectively. The HRs for all-cause mortality in hypertensive patients were 1.14 (95% CI:1.07–1.21) for those with 50–75% TTR, 1.92 (95% CI:1.81–2.04) for 25–50% TTR, and 2.97 (95% CI: 2.80–3.16) for less than 25% TTR, using the > 75% TTR group as the reference [15].

In a population-based cohort study published in 2018, 169,082 individuals with newly diagnosed hypertension, but without a prior history of CV disease, were analyzed from January 1997 to March 2010. The target range, although not including a lower limit of BP, was defined as < 140/90 mm Hg or < 150/90 mm Hg for patients without diabetes or chronic kidney disease that were older than 60 years of age. TTR was calculated using 1.64 million clinical BP readings, with each patient having on average 1.6 BP measurements per year. Patients were followed for 4.9 years on average. Compared to individuals with TTR of 0%, those with TTR durations of 3–5.9 months/year and 6–8.9 months/year demonstrated significantly reduced odds of experiencing CV death, MI, or stroke with adjusted odds ratios (OR) of 0.25 (95% CI:0.21–0.31) and 0.22 (95% CI:0.17, 0.27) respectively. Additionally, increased TTR was associated with decreased risk of heart failure and any CV disease and death [OR comparing 3–5.9 months and 6–8.9 months to 0% TTR, 0.0.37 (95% CI:0.29-0.0.38) and 0.3 (95% CI:0.22–0.42)] [14].

## Impact on Heart Failure Outcomes

A post- hoc analysis of Treatment of Preserved Cardiac Function Heart Failure with an Aldosterone Antagonist (TOPCAT) trial and the Beta-Blocker Evaluation of Survival Trial (BEST) was performed to assess the prognostic value of time spent within the BP target range in patients with HF. TTR, defined as systolic BP between 120 and 130 mm Hg, was calculated for each patient through RLI. The study included a total of 4,789 patients, and the cumulative incidence of the primary endpoint, defined as CV death or heart failure hospitalization, was assessed. The highest quartile of TTR was associated with a 29% reduced risk of the primary endpoint [HR 0.71 (95% CI: 0.60–0.82)], heart failure hospitalization [HR 0.70 (95% CI:0.58–0.82)], and all-cause mortality [HR 0.69 (95% CI:0.58–0.83)] [20].

Similarly, Huang et al. conducted a secondary analysis of TOPCAT alone. Using RLI, the study calculated the systolic BP TTR, with target range set between 110 and 130 mm Hg. After adjusting for factors including mean systolic BP, the analysis found that a one-standard deviation increase in TTR (38.3%) was linked to a lower risk of the primary composite endpoint of CV death, aborted cardiac arrest or HF hospitalization, with HR of 0.81 (95% CI:0.73–0.90). Additionally, improvements in TTR were associated with reductions in all-cause mortality HR 0.81(95% CI:0.73–0.90), CV death HR 0.78 (95% CI:0.68–0.90), and heart failure hospitalization HR 0.85 (95% CI: 0.74–0.97). These relationships were particularly marked in younger individuals, suggesting an age-dependent effect. This was shown in subgroup analyses, with (P_interaction_ = 0.028) [21].

## Impact on Incident Atrial Fibrillation

A post hoc analysis of SPRINT focused on the prognostic value of systolic BP TTR for incident atrial fibrillation (AF) in patients with hypertension. Among 7,939 participants, 187 incident AF cases occurred during follow-up. After adjusting for various factors, including treatment arm, a 10% increase in systolic BP TTR was independently associated with a 7% lower risk of incident AF [HR 0.93 (95% CI:0.88–0.97)]. There was a linear and inverse relationship observed between systolic BP TTR, and incident AF [22].

## Renal Denervation and its Impact on Time in Target Range

The impact of catheter-based renal denervation on systolic BP TTR and its subsequent association with CV outcomes among individuals with uncontrolled hypertension was assessed using the Global SYMPLICITY Registry. The study included 3,077 patients with uncontrolled hypertension. Both office and ambulatory systolic blood pressure measurements were utilized to calculate TTR, with target ranges set at ≤ 140 mm Hg for office SBP and ≤ 130 mm Hg for ambulatory SBP. TTR was derived using RLI method interpolating successive BP measurements from baseline through the first 6 months post-RDN. This initial 6-month period was used to predict CV events occurring from 6 to 36 months post-procedure. The findings demonstrated that a 10% increase in TTR during the first 6 months was associated with significant reductions in MACE (composite of CV death, MI or stroke) by 15% [HR 0.85(95% CI:0.79–0.91)], CV death by 11% [HR 0.89 (95% CI:0.81–0.97)], MI by 15% [HR 0.85(95% CI:0.75–0.98)], and stroke by 23% [HR 0.77 (95% CI:0.68–0.88)] over the subsequent 30 months [23].

## Impact on Kidney Outcomes

The above-mentioned study by Buckley et al. investigated SBP TTR in relation to adverse kidney events as well as CV events using SPRINT and ACCORD data. Adverse kidney events were defined as a composite outcome, including chronic dialysis, kidney transplant, serum creatinine levels exceeding 3.3 mg/dL, sustained estimated glomerular filtration rate less than 15 mL/min/1.73 m^2^, and a sustained decline in eGFR of more than 40%. Compared to the 0% TTR group, participants with > 0% to 43% TTR showed a 41% risk reduction with a HR of 0.59 (95% CI:0.44–0.80). This substantial renal protection was consistent in the 43% to < 70% TTR group [HR 0.60 (95% CI:0.44–0.82)] and further improved in the 70% to < 100% TTR group [HR 0.56 (0.40–0.78)]. The greatest benefit was observed in participants with 100% TTR, who experienced a 65% reduction in risk [HR 0.35 (95% CI:0.21–0.59)]. The majority of event reduction occurred in the > 0% to 43% TTR category, demonstrating that even modest BP control yields substantial benefits in reducing adverse kidney events, with further risk reduction achieved at higher levels of BP control. The authors postulate that renal autoregulation plays a role in the observed data [17].

## Impact on Dementia

Hypertension is a recognized risk factor for dementia. Recent meta-analyses and clinical trial data demonstrate that BP lowering was associated with a decreased risk of dementia [24–27]. However other evidence from a comprehensive meta-analysis of seven population-based cohorts revealed a U-shaped association between BP and dementia risk, which raises concerns over BP lowering in the older population [28]. Therefore, clarity over the effects of BP control strategies is crucial. In light of the conflicting evidence, and the fact that BP is a continuous measure that can vary, TTR has emerged as a novel metric of BP management which allows us to look at data differently.

Recently, Li et al. conducted a post hoc analysis of the SPRINT MIND trial (Systolic Blood Pressure Intervention Trial Memory and Cognition in Decreased hypertension), a clinical trial that examined intensive (< 120 mm Hg) versus standard (< 140 mm Hg) SBP lowering interventions in individuals with hypertension and high cardiovascular risk but without a history of stroke or dementia [29]. TTR was calculated from baseline to month 3 using 110 to 130 mm Hg and 120 to 140 mm Hg as target range for the intensive and standard groups, respectively and notably patients were grouped into tertiles of TTR (0% to < 43%, 43% to < 76, and %76% to 100%). Dementia risk was reduced for every tertile increase in systolic BP TTR, however no association was seen with either mild cognitive impairment or the composite endpoint of probable dementia and cognitive impairment. Similarly, another post-hoc analysis by Huang et al. where patients were grouped into quartiles of TTR (0% to < 27%, 27% to < 56%, 56% to < 80%, and 80% to 100%), an independent association between lower TTR and increased risk of probable dementia was observed whereby the risk of probable dementia and the composite outcome increased 18% per quartile decrease of TTR, and it’s important to note that this association was consistent among both treatment arms (standard vs intensive). These findings propose that the elevated risk observed with lower TTR would not be attributable to the distinct BP control strategies employed in SPRINT.

## Wearable Devices and Potential Impact on Time in Target Range Assessment

The integration of technology into daily routines is becoming widespread. For example, wearable devices have increased the availability of health information, enabling both patients and healthcare providers to access real-time data and insights [30–32]. Although BP is conventionally assessed preferably in the ambulatory setting using oscillatory devices [33, 34], wearable devices now offer additional data points to evaluate not just heart rate and rhythm but BP [35]. Novel devices that can measure BP in real time without cuffs, by using metrics such as pulse transit time, pulse wave velocity, and pulse arrival time may be well suited to assess BP TTR [35–37]. One caveat to the use of wearable devices to assess BP is that most of these devices remain in the prototype stage of development, are not yet commercially available, and have not undergone clinical validation or comparison with traditional BP measurement methods [37]. Integrating SBP TTR with wearable cuffless devices may significantly enhance BP management. These devices provide clinicians with real-time BP data and detailed insights into daily, weekly, and monthly BP patterns. This continuous monitoring has the potential to allow for more effective and timely treatment adjustments, ultimately reducing the future risk of developing CV disease. By mirroring the approach used in diabetes management with continuous glucose monitoring, wearable BP devices would enable precise control and better overall patient outcomes [38–40].

## Conclusion and Future Perspectives

**Fig. 1:**
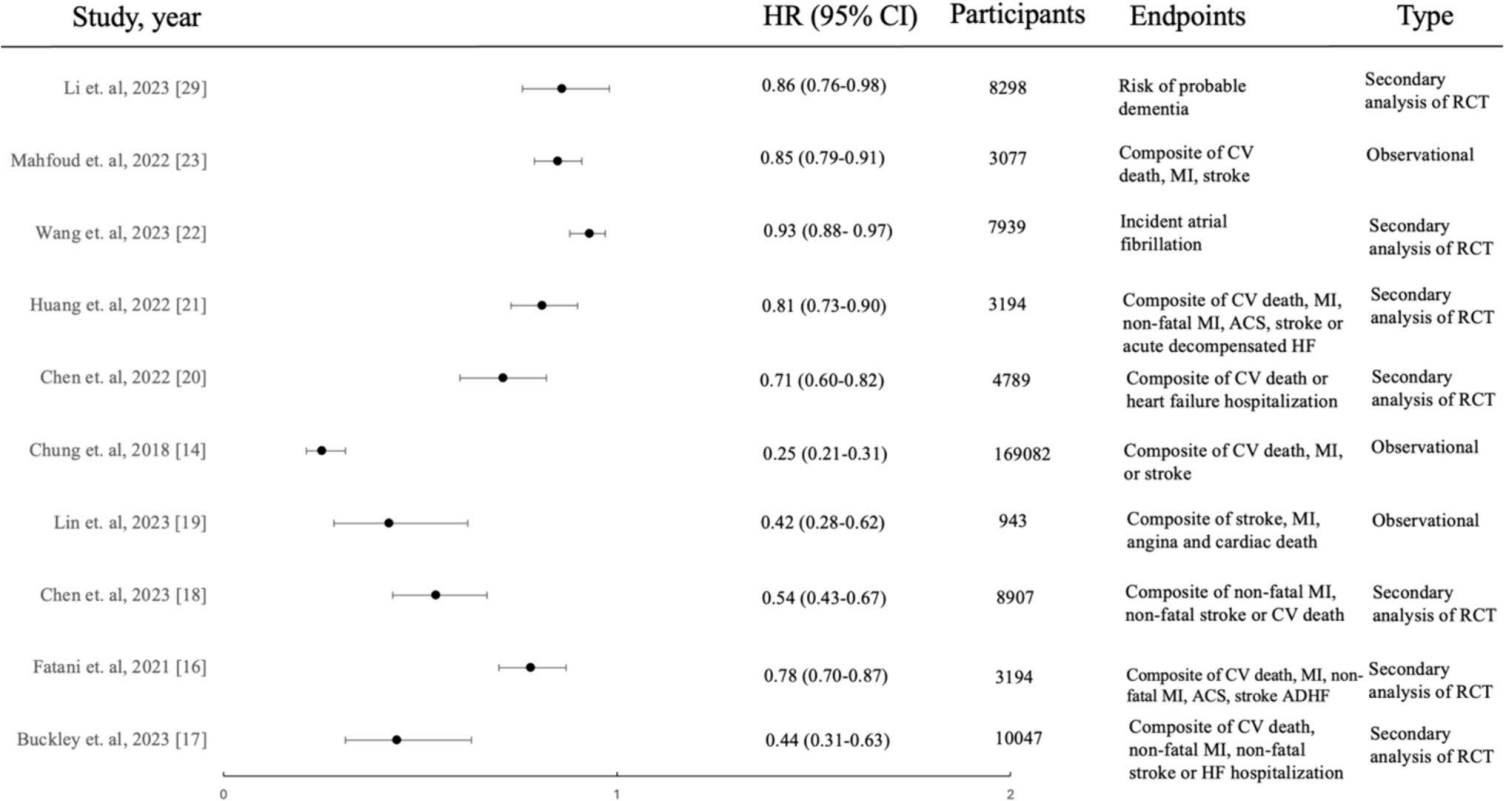
Forest plot of HRs and 95% CIs for various outcomes from blood pressure time in target range analyses. For the studies of Mahfoud et. al and Chen et. al (2023), Wang et.al, HRs were computed for 10% increase in TTR. For the study by Chung, TTR for 3–5.9 months/year was used. For the studies by Chen et. al, Lin et. al and Buckley et.al the highest quartile of TTR was used. Otherwise, for the rest of the studies, HRs calculated for 1 standard deviation increase in TTR

Blood pressure TTR is an emerging concept with potential clinical implications and use. Increasing evidence suggests a positive correlation between extended durations spent within specific target ranges and a reduction in CV morbidity and mortality (Fig. 1). However, challenges remain in fully understanding the complexities of the TTR framework.

Healthcare systems often operate based on metrics, and TTR could be utilized as a quality metric within electronic medical records. Despite this potential application, the literature currently lacks clear definitions for optimal target ranges. While adherence to the target range near 100% is associated with improved cardiac outcomes, the incremental benefits of varying durations within the range, such as between 30 and 60% adherence, are not well-defined. Additionally, there is ongoing debate regarding the reliability of office versus home BP measurements for calculating TTR. The majority of TTR studies rely on office BP readings from clinical trials, which are known to differ from readings taken at home.

Finally, no randomized clinical trials use BP TTR as a pre-specified endpoint. For clinicians to fully embrace BP TTR as a treatment paradigm, clinical trials in which TTR is assessed in a prospective manner will be needed.

TTR holds promise as a way for more precise management of hypertension, however more extensive study is needed.

## Data Availability

No datasets were generated or analysed during the current study.

## Abbreviations

*ACS*: Acute coronary syndrome
*CI*: Confidence Interval
*CV*: Cardiovascular
*HF*: Heart failure
*HR*: Hazard Ratio
*MI*: Myocardial infarction
*RCT*: Randomized Clinical Trial
